# Personal

**Published:** 1911-05

**Authors:** 


### PERSONAL

Dr. Robert F. Sheehan, of Buffalo, has been appointed an assistant surgeon in the United States Navy.

---

Dr. John B. Deaver, of Philadelphia, was the guest of honor at the sixty-fifth annual banquet of the Northern Medical Association of Philadelphia, held on Tuesday evening, April 11, 1911. Dr. Thomas Shriner was toastmaster, and in addition to his speech, and that of Dr. Deaver, addresses were made by Dr. D. Riesman and Dr. H. C. Paist.

---

Dr. Arthur C. Schaefer, assistant health commissioner of Buffalo, has been appointed to the medical staff of the 74th regiment, ranking as second lieutenant.

---

Dr. Charles S. Meahl, of Buffalo, has been appointed examiner of the insane by Health Commissioner Fronczak. The position, which is a new one, pays $1,000 a year.

---

Dr. Winford H. Smith has been appointed superintendent of the Johns Hopkins Hospital, vice Dr. Henry M. Hurd, resigned, who will become secretary of the institution and advisor of the board of trustees. Dr. Smith is a Hopkins man, class of 1903. He is experienced in hospital organisation and management. Since February 1, 1909, he has been the general medical superintendent of Bellevue and allied hospitals of New York, including Bellevue, Fordham, Harlem and Gouverneur Hospitals.

